# Weyl fermions and spin dynamics of metallic ferromagnet SrRuO_3_

**DOI:** 10.1038/ncomms11788

**Published:** 2016-06-08

**Authors:** Shinichi Itoh, Yasuo Endoh, Tetsuya Yokoo, Soshi Ibuka, Je-Geun Park, Yoshio Kaneko, Kei S. Takahashi, Yoshinori Tokura, Naoto Nagaosa

**Affiliations:** 1 Institute of Materials Structure Science, High Energy Accelerator Research Organization, 1-1 Oho, Tsukuba 305-0801, Japan; 2 Department of Materials Structure Science, School of High Energy Science, Graduate University for Advanced Science, Tsukuba 305-0801, Japan; 3 RIKEN Center for Emergent Matter Science, Wako 351-0198, Japan; 4 Department of Physics and Astronomy, Seoul National University, Seoul 151-747, Korea; 5 Center for Correlated Electron Systems, Institute for Basic Science, Seoul 151-747, Korea; 6 Department of Applied Physics, University of Tokyo, Tokyo 113-8656, Japan

## Abstract

Weyl fermions that emerge at band crossings in momentum space caused by the spin–orbit interaction act as magnetic monopoles of the Berry curvature and contribute to a variety of novel transport phenomena such as anomalous Hall effect and magnetoresistance. However, their roles in other physical properties remain mostly unexplored. Here, we provide evidence by neutron Brillouin scattering that the spin dynamics of the metallic ferromagnet SrRuO_3_ in the very low energy range of milli-electron volts is closely relevant to Weyl fermions near Fermi energy. Although the observed spin wave dispersion is well described by the quadratic momentum dependence, the temperature dependence of the spin wave gap shows a nonmonotonous behaviour, which can be related to that of the anomalous Hall conductivity. This shows that the spin dynamics directly reflects the crucial role of Weyl fermions in the metallic ferromagnet.

## Abstract (web summary)

Whilst Weyl fermions have characteristic effects in magnetotransport measurements in condensed matter systems, their effects on other properties remain unexplored. Here, the authors use neutron Brillouin scattering to evidence the interaction of Weyl fermions with the spin wave dynamics in SrRuO_3_.

## Graphical abstract

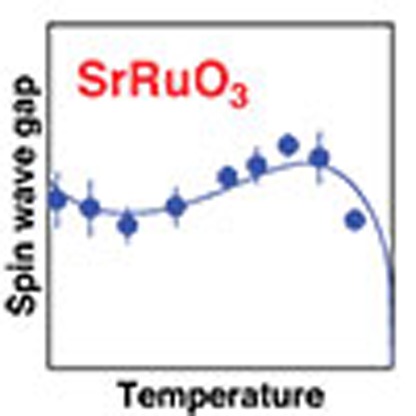

The spin dynamics in magnets is an important subject studied for many decades. Early studies are based on the spin Hamiltonians where the electronic states are indirectly reflected in the magnetic parameters^1^. Recently, microscopic understanding starting from the electronic band structures has become a realistic target with the help of advanced first-principles calculations^2^,^3^,^4^.

In ferromagnets, repulsive Coulomb interaction between electrons results in an exchange splitting *J*_ex_ between bands with up (↑) and down (↓) spins, which produces spontaneous magnetization, as shown in Fig. 1a. Therefore, the band crossings *ɛ*_*n*↑_(**k**)=*ɛ*_*m*↓_(**k**) (*n*≠*m*) between the different bands (*n*th and *m*th bands), where *ɛ*_*n*↑_(**k**) and *ɛ*_*m*↓_(**k**) are the momentum (**k**) dependence of the energy bands of the corresponding states, occur on two-dimensional surface in the three-dimensional momentum space. In the presence of spin–orbit interactions, *λ*_1_(**k**) and *λ*_2_(**k**), that mix different spin components and give the off-diagonal matrix elements, the band crossings turn into anti-crossings with the gap as indicated by the red curve in Fig. 1a. More explicitly, the effective Hamiltonian reads


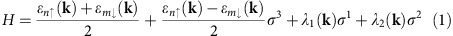


with 2 × 2 Pauli matrices *σ*=(*σ*^1^,*σ*^2^,*σ*^3^), and the gap is given by 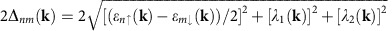. This gap closes when the three coefficients of the Pauli matrices *σ*'s are zero in equation (1), which can be satisfied by tuning the three components of the momentum **k** at some **k**_0_ in three dimensions. Figure 1b shows this situation, where the energy dispersions at each fixed *k*_*z*_ and the gap closes at **k**=**k**_0_. Expanding equation (1) with respect to **k**–**k**_0_, one obtains *H*=*ηv*(**k**–**k**_0_)·*σ* with the momentum **k** being appropriately redefined. Here *v* is the velocity and *η*=±1 determines the chirality. This is nothing but the Weyl fermion and **k**_0_ is called Weyl point, which is ubiquitous in magnets as long as the band overlaps of both spin components are finite.

It has been pointed out that the band crossing of the form in equation (1) is of crucial importance for the Berry curvature; it acts as a source or sink (monopole) of the emergent magnetic field **b**(**k**). More explicitly, 
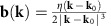
 as shown in Fig. 1c, and the flux integral over the surface enclosing **k**_0_ is 2*πη*. This emergent magnetic field gives the anomalous velocity and Hall current in the presence of the external electric field, which is the origin of the intrinsic anomalous Hall effect in ferromagnets: that is, *σ*_*xy*_ can be written as the integral of the Berry phase curvature (the gauge field) over the occupied electronic states^5^,^6^,^7^,^8^. The anomalous Hall effect emerging in the ferromagnetic phase of SrRuO_3_ has been analysed from this viewpoint, where *σ*_*xy*_ shows nonmonotonous dependence on the magnetization. We note that this anomalous Hall effect can be well reproduced by a first-principles calculation. In the ferromagnetic phase of SrRuO_3_, also a tight-binding model reveals that a number of Weyl points are produced in the first Brillouin zone by the above-mentioned process^9^. Therefore, the origin of the Berry curvature resulting in the nonmonotonous *σ*_*xy*_ in SrRuO_3_ is Weyl fermions as magnetic monopoles^6^,^7^,^8^,^9^,^10^. The optical Hall conductivity *σ*_*xy*_(*ω*) in the terahertz region also supports the presence of Weyl fermion within the range of 5 meV near the Fermi energy^11^.

Recently, several materials have been theoretically proposed and experimentally studied, where Weyl fermions are located exactly at the Fermi energy and govern the transport phenomena, that is, Weyl semimetals^12^,^13^,^14^,^15^,^16^,^17^,^18^. For example, the large magnetoresistance is discussed from the viewpoint of chiral anomaly. We note that the previous studies are, however, restricted mostly to the transport properties, and the influence of Weyl fermions on other phenomena are unexplored.

In this paper, we report the fingerprint of Weyl fermions and magnetic monopoles in the spin dynamics of SrRuO_3_. It has been recognized that SrRuO_3_ is a rare example of 4d band ferromagnetic metal with a pseudo-cubic perovskite crystal structure, and the ferromagnetism has been extensively studied^19^,^20^,^21^. Recently, a large spin wave gap was observed in SrRuO_3_ (ref. 22), whereas La_0.8_Sr_0.2_MnO_3_, a ferromagnet having a similar pseudo-cubic structure, showed no spin wave gap^22^. Although the large magnetic anisotropy in SrRuO_3_ is one of remarkable features, no anomaly in the temperature dependence of the magnetic anisotropy has been found in the static properties^19^,^20^,^21^,^23^. We show that Weyl fermions play a crucial role in the spin dynamics by measuring the temperature dependence of spin waves in SrRuO_3_.

## Results

### Spin waves observed by neutron Brillouin scattering (NBS)

**Figure 2:**
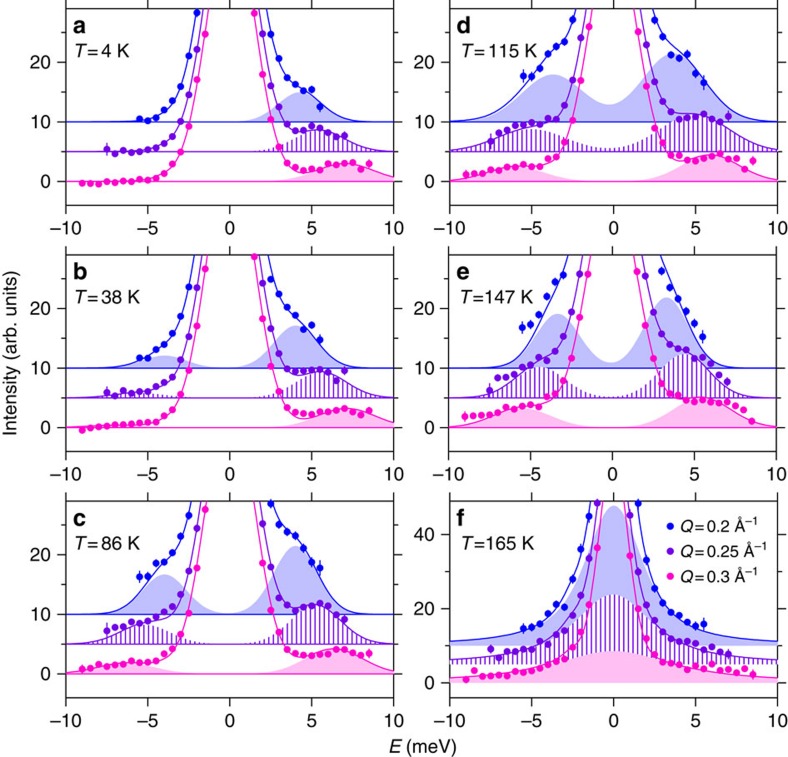
Background subtracted INS spectra in SrRuO_3_. Observed spectra are displayed as a function of *E* at several *Q* values (0.2, 0.25, 0.3 Å^−1^) for selected *T* (**a**: 4 K, **b**: 38 K, **c**: 86 K, **d**: 115 K, **e**: 147 K, **f**: 165 K). Spectra are shifted by a constant to aid the eye. The vertical bars represent statistical errors determined from the square root of neutron counts. The solid lines are fitted curves to the observed spectra, and the shaded areas are magnetic components.

**Figure 3:**
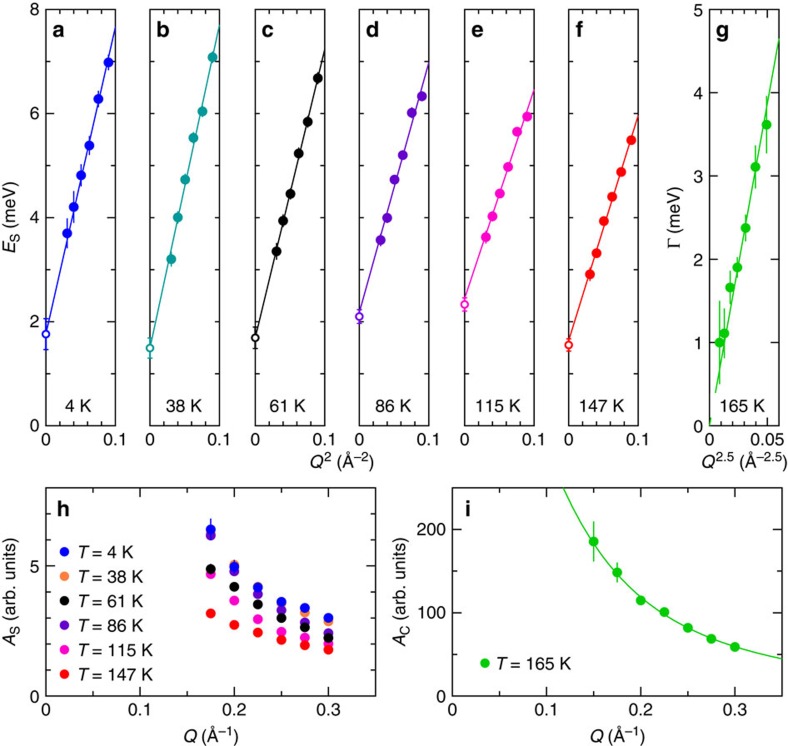
Parameters in scattering function for spin waves and critical scattering in SrRuO_3_. (**a**–**f**) Spin wave energy *E*_S_ and (**h**) intensity *A*_S_(*Q*) are plotted in *Q* for selected *T* below *T*_C_=165 K. The lines in (**a**–**f**) are fitted to the quadratic dispersion relation, and the open circles at *Q*=0 Å^−1^ are the spin wave gap *E*_g_ obtained by the fit. (**g**) Energy width Γ and (**i**) intensity *A*_C_(*Q*) of the paramagnetic scattering at *T*=*T*_C_ are plotted in *Q*. The lines in **g** and **i** are the fitted curves to the dynamical scaling law. The vertical bars represent statistical errors, some of them are smaller than the sizes of the marks.

We have measured small momentum magnetic excitations in a polycrystalline sample of SrRuO_3_ by NBS experiments, namely inelastic neutron scattering (INS) experiments near the forward direction. In case that a sizable single crystal is not available, NBS is the most appropriate magnetic INS method for studying the spin dynamics. The state-of-the-art instrument (High-Resolution Chopper Spectrometer, HRC) at intense pulsed neutron scattering facilities like J-PARC (Japan Proton Accelerator Research Complex) has provided an opportunity for the present study approaching the kinematical constraint of the neutron scattering by providing high-energy neutrons of sub eV region. Typical data of the observed INS spectra are shown in Fig. 2, and were analysed by using the scattering functions *I*(*Q*,*E*) expressed as follows:


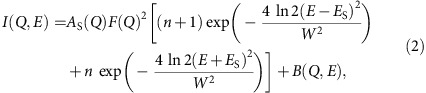



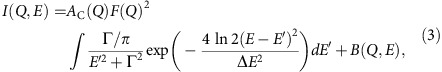


where *E* and *Q* is the energy and momentum transfers of neutrons, respectively. Equation (2) presents the spectrum for the spin waves with energy *E*_S_ at temperature *T* below the ferromagnetic transition temperature *T*_C_ (=165 K), and equation (3) corresponds to the paramagnetic scattering with the energy width Γ at *T*=*T*_C_. The magnetic form factor of spin component can be approximated to be *F*(*Q*)=1 in the present small *Q* range. *A*_S_(*Q*) and *A*_C_(*Q*) represent the other *Q* dependence in the scattering intensity. The thermal population factor is given by *n*+1=[1–exp(−*E*/*k*_B_*T*)]^−1^ with the Boltzmann constant *k*_B_. Each scattering function is the form convoluted with the instrumental resolution, and contains the elastic scattering component *B*(*Q*,*E*). Δ*E* is the energy resolution and *W* is the energy width of spin waves determined from the instrumental resolution. Δ*E* and *B*(*Q*,*E*) are determined experimentally. The quantities, *A*_S_, *A*_C_, *E*_S_ and Γ, are determined by the standard least square fitting procedure, as shown in Figs 2 and 3.

**Figure 4:**
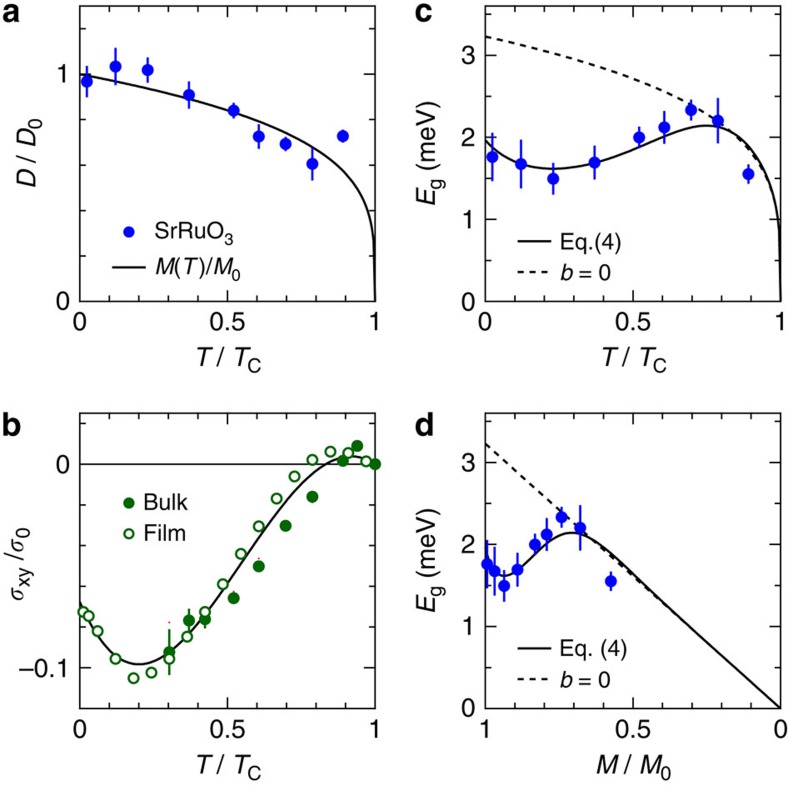
Stiffness constant and spin wave gap in SrRuO_3_. (**a**) Stiffness constant *D*, (**b**) anomalous Hall conductivity *σ*_*xy*_/*σ*_0_ with σ_0_=9.9 × 10^2^ Ω^−1^ cm^−1^, (**c**) spin wave gap *E*_g_ as a function of *T*/*T*_C_ and (**d**) *E*_g_ as a function of *M*(*T*)/*M*_0_. The vertical bars in **a** and **c** represent the statistical errors of the measured values. The solid line in **a** is the measured curve of *M*(*T*)/*M*_0_. The experimental data of *σ*_*xy*_(*T*) for the bulk sample and those for the film sample scaled to the bulk samples are plotted with an empirical curve (solid line) to express *σ*_*xy*_(*T*) for both samples in **b**, where the vertical bars represent experimental errors. The solid lines in **c** and **d** are the fit to equation (4) with *a*=3.2 meV and *b*=−9.5, and the dashed lines in **c** and **d** are the calculated curves with *a*=3.2 meV and *b*=0, by using the empirical curve for *σ*_*xy*_. It should be noted that *D*(*T*) and *E*_g_(*T*) are proportional to magnetization *M*(*T*) in the strong correlation limit.

The spin wave energies *E*_S_ can be well fitted to the simple dispersion relation for ferromagnetic spin waves *E*_S_(*Q*)=*E*_g_+*DQ*^2^, which is clearly extrapolated to the finite spin wave gap *E*_g_∼2 meV, as shown in Fig. 3a–f. The peak intensities *A*_S_(*Q*) in Fig. 3h increase as *Q* gets reduced, which suggests that the magnetic excitations contain another component, that is, orbital moment, other than spin. The large *E*_g_ emerges due to the spin–orbit interaction as described below. We note that the increase in *A*_S_(*Q*) at low *Q* can be in accord with the spreading of the orbital component in real space. As shown in Fig. 4a, *D* decreases as *T* increases, that is a normal behaviour observed in both localized electron systems and itinerant electron systems^24^. On the other hand, *E*_g_ shows nonmonotonous thermal evolution. The finite value of *E*_g_ indicates an internal magnetic field acting on the system.

## Discussion

The *T* dependence of *E*_g_ is nonmonotonous and clearly differs from that of the spontaneous magnetization *M*. A magneto-optical measurements showed the ferromagnetic resonance (FMR) frequency at 250 GHz almost independent of *T* at *T*<80 K (ref. 23); this also differs from *M*(*T*). Both *E*_g_ and the FMR frequency show the anomalous *T* dependence. The observed *E*_g_=1.5–2.1 meV at low *T* including the error bars is of the comparable magnitude as *E*_g_∼1 meV estimated by the FMR frequency of 250 GHz. The anisotropy energy of *K*∼12 T estimated by the bulk magnetization^21^ is comparable to these values. At zero temperature, 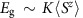 is given both in the strong and weak correlation cases; if we use the value 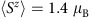 for a powder sample^25^, we obtain *E*_g_∼1 meV. The dipole interaction can also contribute to the spin wave gap at the zone centre, which is directly observed by the FMR and Brillouin light scattering. Such gap is 0.03 meV for yttrium iron garnet film^26^, and we anticipate it to be smaller for SrRuO_3_ having smaller magnetic moment than the yttrium iron garnet film. It should be noted that, in the NBS using a polycrystalline sample, *E*_g_ is extrapolated from the dispersion observed at finite *Q* by mapping onto the simple spin Hamiltonian neglecting such a small dipole interaction.

The nonmonotonous *T* dependence of the anomalous Hall conductivity *σ*_*xy*_ of SrRuO_3_ was discussed from the viewpoint of Weyl fermions, that is, magnetic monopoles in the momentum space^6^,^8^,^10^. Similarly, Weyl fermions are expected to produce the nonmonotonous *T* dependence of the spin dynamics as well. As discussed below, *E*_g_ is expected to be fitted by the following formula;


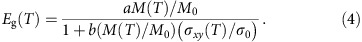


Here, *σ*_0_=*e*^2^/*ha*_0_=9.9 × 10^2^ Ω^−1^ cm^−1^ (*a*_0_ being the lattice constant of SrRuO_3_) is used as a normalization factor for *σ*_*xy*_. As shown in Fig. 4c,d, the nonmonotonous *T* dependence of *E*_g_ can be well reproduced by using the parameter of *a*=3.2 meV and *b*=−9.5. For *M*(*T*)/*M*_0_ and *σ*_*xy*_(*T*)/*σ*_0_, we used the experimental results (see the Methods for details).

Now we sketch the theoretical background of equation (4) (see the Methods for details). The spin wave at the zero momentum is described by the action obtained by integrating over the electrons as (ref. 27) 


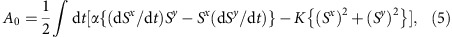


where the first term with the coefficient *α* is the integrand represent of the Berry phase, whereas the second term is the spin anisotropy energy. Then spin wave gap *E*_g_(*T*) is given by the ratio *K*/*α*. The main idea is that the Weyl fermions contribute resonantly to *α* as in the case of *σ*_*xy*_ because of the small energy denominator, although the former takes the finite value *α*_0_ even without the spin–orbit interaction, whereas the latter comes solely from the spin–orbit interaction (see the Methods for details). Assuming a single Weyl fermion near the Fermi energy, as suggested by the terahertz optical spectroscopy^11^, there is a one-to-one correspondence between the spin operators and current operators. Therefore, the contribution *α*_1_ to the coefficient *α* from the Weyl fermion is given by *λσ*_*xy*_ with *λ* being a constant. Since 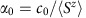 (see the Methods for details), we obtain 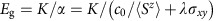, which is equivalent to equation (4). Here the temperature-independent anisotropy energy *K* is assumed, which is justified down to *T*∼40 K by the magnetization curve measurement^28^.

To conclude, the nonmonotonous temperature dependence of the spin wave gap is unveiled by NBS in SrRuO_3_, which is related to its nonmonotonous anomalous Hall effect through the Weyl fermions near the Fermi energy. This result has revealed the connection between the transport and dynamical magnetic properties through the enhanced spin-orbit coupling effect. NBS employed here has the large potentiality. It can provide the detailed information of the generalized magnetic susceptibility in the small *Q*-region in addition to the energy of the magnetic excitations, which we focused in the present paper. NBS will thus provide a powerful method to solve unsettled issues in dynamical magnetism, including a role of orbital component in the magnetic fluctuation.

## Methods

### Materials

**Figure 5:**
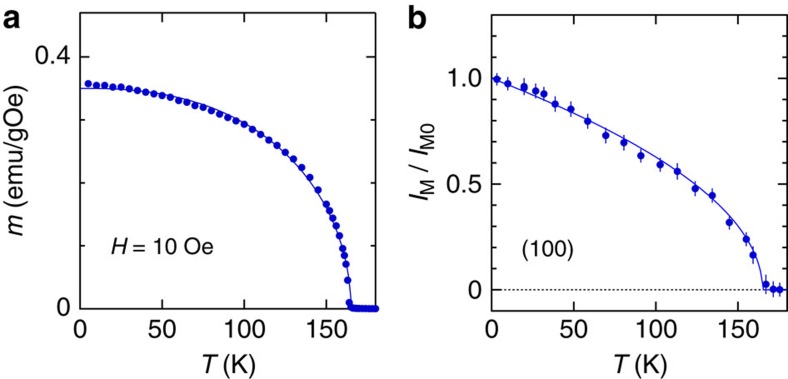
Determination of ferromagnetic transition temperature *T*_C_ and critical exponent *β*. (**a**) The ferromagnetic transition temperature *T*_C_ (=165 K) is estimated from the temperature dependence of the low-field magnetization. (**b**) The temperature dependence of magnetic Bragg scattering at (100) is well fitted to *I*_M_(*T*)=*I*_M0_(1–*T*/*T*_C_)^2*β*^, and the critical exponent is determined to be *β*=0.25±0.01 by using *T*_C_=165 K.

The sizable single crystals of SrRuO_3_ necessary for INS experiments have never been synthesized so that the spectroscopic studies must be performed using powder (polycrystalline) samples. We prepared polycrystals of SrRuO_3_ (73 g) by the well-established method of synthesis^25^, and the high quality of the sample was confirmed by our subsequent bulk measurements. The crystal structure with a single phase was confirmed by the X-ray diffraction. The magnetization measurement under the low magnetic field (*H*=10 Oe) is well fitted to *m*(*T*)=*m*_0_(1–(*T*/*T*_C_)^*a*^)^*b*^, as shown in the inset of Fig. 5a, and the ferromagnetic transition temperature was determined to be *T*_C_=165 K. This value of *T*_C_ is the highest in the powder forms and comparable to that of the single-crystal forms.

The elastic neutron scattering from the magnetic Bragg reflection at (100) was observed on the HRC (see below) with *E*_i_=12.5 meV. The observed intensity is the sum of the *T*-dependent magnetic component *I*_M_ and the constant nuclear component *I*_N_, and thus, well fitted to *I*(*T*)=*I*_M_+*I*_N_=*I*_M0_(1–*T*/*T*_C_)^2*β*^+*I*_N_ with the above-obtained value of *T*_C_=165 K by parameterizing *β*, *I*_M0_ and *I*_N_, as shown in Fig. 5b, where *I*_M_/*I*_M0_=(*I*(*T*)–*I*_N_)/*I*_M0_ is plotted. The obtained critical exponent *β*=0.25±0.01 agrees well with that from the early neutron diffraction experiment^29^. Therefore, we used the *T* dependence of the saturation magnetization, *M*(*T*)/*M*_0_=(1–*T*/*T*_C_)^*β*^ with these values of *T*_C_ and *β* for the analysis of *D*(*T*) and *E*_g_(*T*). The stiffness constant *D*(*T*) at *T*=4–130 K was fitted with *D*_0_(1–*T*/*T*_C_)^*β*^ and *D*_0_ was obtained to be 62 meV.

For the transport measurement, the powder sample of SrRuO_3_ obtained in the same batch as that for the INS experiments was sintered by high-pressure spark plasma sintering and patterned in Hall bar geometry using conventional photo-lithography and Ar ion dry-etching. The Hall resistivity *ρ*_H_ was measured together with the longitudinal and transverse resistivities, *ρ*_*xx*_ and *ρ*_*yx*_, as a function of *T* under an applied magnetic field. The anomalous resistivity *ρ*_*yx*_ was determined after subtracting the ordinary Hall contribution from the measured *ρ*_H_, and the transverse conductivity *σ*_*xy*_ was determined as *ρ*_*yx*_/(*ρ*_*xx*_^2^+*ρ*_*yx*_^2^)≅*ρ*_*yx*_/*ρ*_*xx*_^2^, and plotted in Fig. 4b as the bulk sample. The error bars of *σ*_*xy*_ for the bulk sample are large at low temperatures because of very small values of resistance. On the other hand, the error bars of *σ*_*xy*_ for the film sample are relatively small because of high resistance and well-defined shape of the Hall bar^10^. We also plotted *σ*_*xy*_ for the film sample^10^, which was scaled to the bulk data by a factor of 0.61. The reduction factor of 0.61 was determined by minimizing the difference (*χ*^2^) between the bulk data and the film data. An empirical formula was established to express *σ*_*xy*_(*T*) for both the film and the bulk samples, as indicated with the solid line in Fig. 4b. For the analysis of *E*_g_(*T*), we used the empirical formula for *σ*_*xy*_(*T*)/*σ*_0_, where *σ*_0_=*e*^2^/*ha*_0_=9.9 × 10^2^ Ω^−1^ cm^−1^ with *a*_0_=3.9 Å being the atomic distance, *e* being the elementary charge and *h* being the Planck's constant.

### NBS and data analysis

To observe ferromagnetic excitations in a polycrystalline sample, we have applied the NBS method by using the HRC installed at the Materials and Life Science Experimental Facility, J-PARC^30^. On the HRC, neutron beams extracted from the pulsed neutron source are monochromatized by a Fermi chopper (the incident neutron energy (*E*_i_) is selected) and are incident upon the sample. Scattered neurons, of which energy transfer (*E*) is determined from the time-of-flight, are collected with a detector system covering the scattering angles continuously. By utilizing high-energy (sub eV) neutrons with the short pulses realizing the high-energy resolution of Δ*E*/*E*_i_∼2% on the HRC, one can perform NBS experiments, that is, INS experiments near to the forward direction like the conventional optical Brillouin scattering method^22^,^30^. Normally, a sizable single crystal is required for INS experiments. However, excitations near to the zone centre, where scattering intensities remain even by the powder average of the dynamical structure factor, can be detected by the NBS.

To measure the spin wave dispersion relation for SrRuO_3_, we selected *E*_i_=100 meV. Then, the transferred energy *E* reaches to higher than 10 meV with the energy resolution Δ*E*=2 and 3.4 meV and the transferred momentum *Q* approaches down to ∼0.15 and 0.175 Å^−1^, respectively, by using the detecting system that covers the scattering angles down to 0.6°. The condition with Δ*E*=3.4 meV provides the peak intensity of the excitation spectrum twice that with Δ*E*=2 meV. Several experimental improvements have made the NBS methods feasible in the practical level for our studies: they include the short-pulse and the high-energy neutrons with higher flux provided by the intense pulsed neutron source as well as the lower background at low angles. Using this new state-of-the-art instrument HRC, we could achieve the data quality equivalent to that by INS measurements with single crystals^22^. These improvements in the instrumentation made it possible for us to get access to the current (*Q*,*E*) space, which is essential for this study. The HRC provides the highest resolution of its class in the (*Q*,*E*) space for the present study.

We measured the INS spectra at *T*=4, 20, 38, 61, 86, 115 and 147 K with the Δ*E*=3.4 meV condition, and at *T*=4, 100, 130 and 165 K with the Δ*E*=2.0 meV condition. It should be noted that the spin wave dispersion relation obtained at *T*=4 K with the Δ*E*=3.4 meV condition was identical to that with the Δ*E*=2.0 meV condition. The scanned data without the sample was defined as the background, which was subtracted from the raw data after normalizing by the exposure time of the data acquisition. The *E* dependence of the background-subtracted intensities were well fitted with equation (2) or (3), 5.

The functional form of the elastic scattering *B*(*Q*,*E*) in equations (2) and (3) is almost described by a Gaussian function centred at *E*=0 meV with Δ*E* being the full-width at half-maximum, however, it has a tiny tail at the negative *E* side. The neutrons are emitted from the neutron source with a time distribution, and the tail originates from the neutrons with higher energies than *E*_i_ emitted later than the time origin of the time-of-flight and transmitted through the time window of the Fermi chopper. The value of Δ*E* and the shape of the tail in *B*(*Q*,*E*) were determined by measuring the shape of the incoherent elastic scattering at low *T* and higher *Q*, where the elastic peak is well separated from the spin wave peaks. As the elastic component consists of the incoherent elastic scattering and the Bragg scattering at the zone centre, *B*(*Q*,*E*) decreases as *Q* increases and becomes constant. The peak value of *B*(*Q*,*E*) can be determined as the peak intensity of the Gaussian component in each spectrum. It should be noted that Δ*E* is independent of *Q*.

The spin wave components could be described by the Gaussian function with the width *W* in equation (2), which was estimated from Δ*E* and the influence of the *Q* resolution via the spin wave dispersion as follows^22^,^31^,


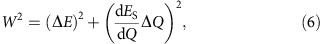


where d*E*_S_/d*Q*=2*DQ*, and Δ*Q*=0.12 Å^−1^ is estimated from the instrumental geometry^22^. The value of *D* in Fig. 4a was used after some iterations. The excitation energy width finer than *W* cannot be determined experimentally. As the tail in *B*(*Q*,*E*) is much smaller than the peak intensity of *B*(*Q*,*E*), the resolution-limited scattering can be approximated to a Gaussian scattering function with the width *W* determined by the instrumental resolution, neglecting the small contribution of the tail. Similar to this, the resolution function with which the Lorentzian component is convoluted for the spectrum at *T*=*T*_C_ can be approximated to a Gaussian scattering function with Δ*E*, as shown in equation (3).

The spin wave energies *E*_S_ decrease upon heating and eventually go to zero at *T*_C_. The spectra also change their nature from the Gaussian function for spin waves to the Lorentzian function centred at *E*=0 meV for the paramagnetic spin fluctuations, as shown in Fig. 2. The scattering function at *T*=*T*_C_ is typical of the critical scattering observed in metallic ferromagnets^32^: the power law Γ=*γQ*^2.5^ clearly holds with *γ*=76 meV Å^2.5^, and the *Q* dependence of intensity *A*_C_(*Q*) is well fitted to the functional form of the critical scattering (*Q*^−2^) convoluted with the instrumental resolution, as shown in Fig. 3g,i. This indicates that the spectra observed at *T*<*T*_C_ are of spin waves.

**Figure 6:**
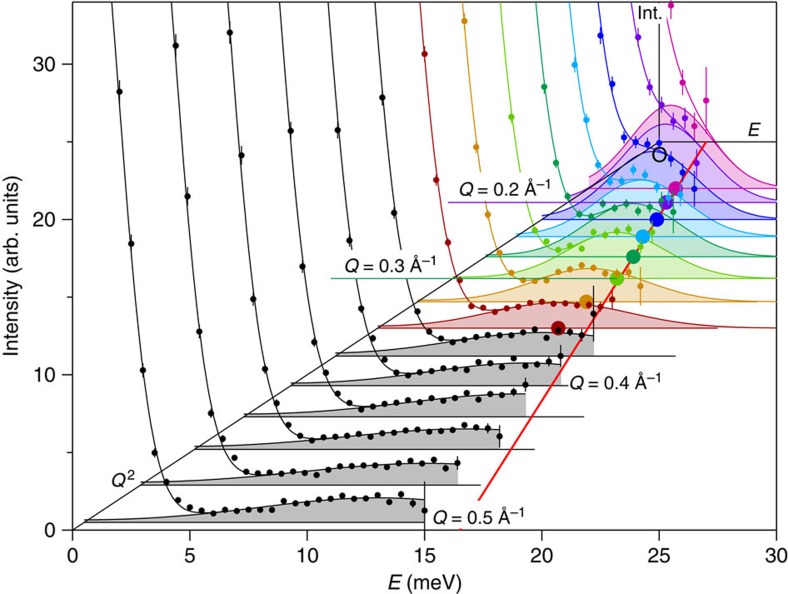
Background subtracted INS spectra in SrRuO_3_ observed at *T*=4 K on the HRC. INS spectra are plotted at *Q*=0.175–0.5 Å^−1^ at a step of 0.025 Å^−1^, which are indicated with small solid circles with vertical bars presenting the statistical errors. Each spectrum was fitted with equation (2) by using the calculated resolution width *W* for *Q*≤0.3 Å^−1^ and by parameterizing *W* for *Q*>0.3 Å^−1^. The solid lines are the fitted curve to the spectrum, and the shaded areas are the spin wave components obtained by the fit. The determined spin wave peak positions are plotted with large circles. The different colours correspond to the different *Q* values. The red line indicates the spin wave dispersion with the *Q*^2^ law determined for *Q*≤0.3 Å^−1^.

In the case of ferromagnetic spin waves, the dispersion relation normally follows the *Q*^2^ law (*E*_S_(*Q*)=*DQ*^2^+*E*_g_) independently of the crystalline orientation in a small *Q* region for cubic or isotropic lattices. In fact, spin waves in the single crystalline sample of the nearly cubic perovskite La_0.8_Sr_0.2_MnO_3_ show the isotropic *Q*^2^ law (*E*_g_=0 meV) at *Q*≤0.3 Å^−1^, although the dispersion curve along [001] bends down to lower energies than the [111] curve at *Q*>0.3 Å^−1^ (ref. 33). The dispersion relation at *Q*≤0.3 Å^−1^ observed by the NBS on the HRC using a polycrystalline sample of La_0.8_Sr_0.2_MnO_3_ was identical to that obtained by using the single crystalline sample^22^. Although the crystal structure of SrRuO_3_ is orthorhombic, similar to La_0.8_Sr_0.2_MnO_3_, it can be approximated to be cubic with the lattice constant of 3.9 Å within the present instrumental resolution. We applied this established experimental technique to the present study of the spin dynamics in SrRuO_3_. Figure 6 shows the INS spectra in SrRuO_3_ observed on the HRC at *Q*=0.175–0.5 Å^−1^ at a step of 0.025 Å^−1^. The spin wave peak positions for SrRuO_3_ were well defined and described by the *Q*^2^ law at *Q*≤0.3 Å^−1^, however, deviated from the *Q*^2^ law at *Q*>0.3 Å^−1^. Furthermore, the spectra became much broader at *Q*⩾0.375 Å^−1^ and the peak positions could not be accurately determined. Therefore, we confirmed that the spin wave dispersion can be approximated to the *Q*^2^ law by using data for *Q*≤0.3 Å^−1^. All our analyses reported here are based on the data collected for *Q*≤0.3 Å^−1^.

### Theoretical analysis

The basic facts about theories of spin wave dispersion and the effects of the Weyl fermions are summarized here.

First, the *T* dependence of the stiffness constant *D* without the spin–orbit interaction has been studied theoretically both in the strong and weak correlation limits. In the strong correlation case, the spin wave is discussed in terms of the Heisenberg spin Hamiltonian 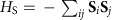. The linearized equation of motion with respect to *S*^+^=*S*^*x*^+*iS*^*y*^ reads


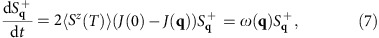


which results in 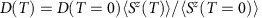, where *Q*=|**q**| for small **q**=(*q*_*x*_, *q*_*y*_, *q*_*z*_) (*Q*≤0.3 Å^−1^ in the present case). On the other hand, in the weak correlation limit, although random phase approximation predicts the diverging *D*(*T*) towards *T*_C_ (ref. 34), the self-consistent renormalization theory predicts the vanishing *D*(*T*) as *T*→*T*_C_ (refs 35, 36). Experimentally, *D*(*T*) for a metallic ferromagnet Ni_3_Al, for instance, decreases to zero as *T* increases to the transition temperature *T*_C_. This behaviour is consistent with both the self-consistent renormalization theory^35^,^36^, and also the Heisenberg model^24^.

In the weak correlation limit, the spin wave dispersion is determined by the generalized spin susceptibility *χ*^+−^(**q**,*ω*) of electrons as given as follows^34^,


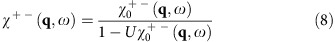


with


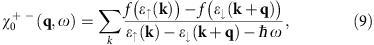


where 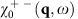 is the generalized spin susceptibility without the electron–electron interaction, *f*(*ɛ*) is the Fermi distribution function and 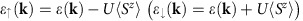 is the energy dispersion of up (down) spin with *U* being the electron–electron interaction. By solving the equation 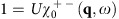, one can obtain the spin wave dispersion *ω*(**q**). Although the detailed form depends on *ɛ*(**k**), one can consider the expansion of *χ*^+−^(**q**,*ω*) with respect to *ω* and **q**. The coefficient *α*_0_ of *ω* is 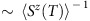, whereas that of **q**^2^ is 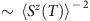 near the transition temperature *T*_C_ (ref. 34).

Next, we consider the effects of the spin–orbit interaction. In the strong correlation limit, it introduces the spin anisotropy term 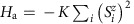, which adds the gap 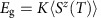 to the spin wave dispersion *ω*(**q**). Although experiments determining the spin wave gap have been often reported, the reports on its temperature dependence are very few. In the case of ferromagnets, the temperature dependence of the spin wave gap is hardly detected, because a ferromagnet having a crystal structure with high symmetry normally shows gapless spin wave. In the case of antiferromagnets, spin wave gap can be enhanced by the exchange interaction *J* as 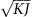. The antiferromagnetic resonance frequency, that is a spin wave gap in antiferromagnet and is also proportional to 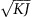, shows a monotonous temperature dependence as a function of the magnetization for insulating antiferromagnets such as MnF_2_ (ref. 37) and MnO (ref. 38). The spin wave gap shows a monotonous temperature dependence as a function of the magnetization even for a metallic antiferromagnet γFeMn (ref. 39).

Equation (9) should be generalized to include the spin–orbit interaction in the electronic band structure,


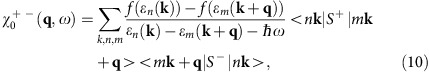


where *n,m* are the band indices including the pseudospin. Equation (10) usually has a complex form, but one can consider the expansion with respect to *ω* and **q** as discussed above. The zero-th order term *χ*^+−^(**0**,0)–*χ*^*zz*^(**0**,0) determines the anisotropy *K*, which is basically independent of *T* near *T*_C_, that is, it can be defined even in the normal phase. On the other hand, when we expand with respect to *ω* as


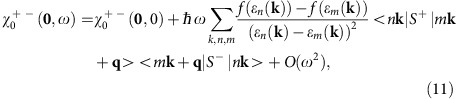


the linear order term in *ω* contains the square of the energy denominator, and is hence sensitive to the Weyl fermions^27^.

Now we discuss how equation (4) is derived theoretically based on the Weyl fermion. The spin dynamics is described by the effective action *A* for the spin fluctuation field *S*^*α*^ obtained by integrating over the electronic degrees of freedom as^27^


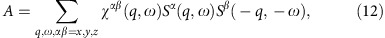


Where 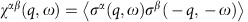 is the correlation function of the conduction electron spin *σ*^*α*^. We are interested in the action *A*_0_ for the uniform component (*q*=0), which is given by equation (5), where the coefficient *α* of the Berry phase term is given by 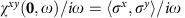, and *K* represents the spin anisotropy energy. Most of the contribution to *α* comes from the bands not largely influenced by the spin–orbit interaction, that is, *α*_0_, and nontrivial contribution *α*_1_ comes from Weyl fermions. As described above, 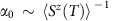. The contribution *α*_1_ from the Weyl fermion is proportional to *σ*_*xy*_, as shown below. The Hamiltonian in equation (1) is rewritten as 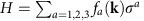, where *f*_*a*_(**k**) is expanded near the band crossing point **k**_0_ as 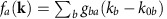. One can relate the current operator *j*_*a*_ and the spin operator *σ*^*b*^ as 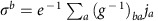 as *j*_*a*_ is obtained by replacing **k** with **k**+*e***A** and taking derivative of the Hamiltonian with the vector potential **A**. By using the above relation between *σ*^*b*^ and *j*_*a*_, 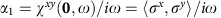 is equal to *λσ*_*xy*_ with the coefficient 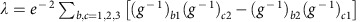. By taking the variational derivative of *A*_0_ in equation (5) with respect to *S*^*x*^ and *S*^*y*^, one can derive the equation of motion and obtain the spin wave gap as 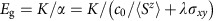, which is equivalent to equation (4) used to fit the data. Thus, the correspondence between *E*_g_ and *σ*_*xy*_ is expected from the fact that the band crossings act as the magnetic monopoles in momentum space. Here we need to assume that *K* is independent of *T* to justify the fitting by equation (4). This fact is consistent with the expectation that the Weyl fermion does not resonantly contribute to *K*, as the energy denominator is *ɛ*_*n*_(**k**)–*ɛ*_*m*_(**k**) in equation (10) for *K*, whereas the expression of *α* in equation (11) contains the energy denominator (*ɛ*_*n*_(**k**)–*ɛ*_*m*_(**k**))^2^.

### Data availability

The authors declare that the data supporting the findings of this study are available within the article.

## Additional information

**How to cite this article:** Itoh, S. *et al*. Weyl fermions and spin dynamics of metallic ferromagnet SrRuO_3_. *Nat. Commun.* 7:11788 doi: 10.1038/ncomms11788 (2016).

## Figures and Tables

**Figure 1:**
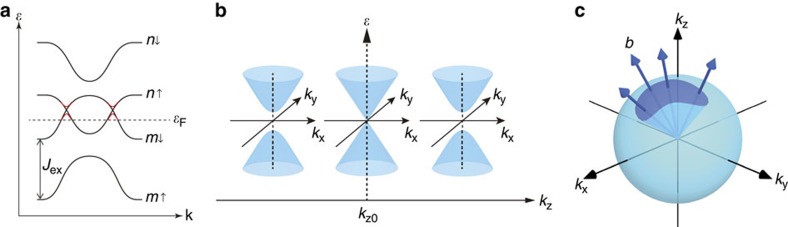
Schematic band structure and Weyl fermion in ferromagnets. (**a**) The energy (*ɛ*) dispersions of up (↑) and down (↓) spin bands with the exchange splitting *J*_ex_. *ɛ*_F_ is the Fermi energy. The band crossings *ɛ*_*n*↑_(**k**)=*ɛ*_*m*↓_(**k**) occur when the overlap of the up and down spin bands are finite. In the presence of the spin–orbit interaction, which mixes the up and down spins, the band crossings turn into the anti-crossings with the gap opening (red curve). (**b**) Band structure near the Weyl point **k**_0_. The band dispersions with fixed *k*_*z*_ are shown in the *k*_*x*_–*k*_*y*_ plane. The gap closes at the Weyl point **k**=**k**_0_. (**c**) Distribution of the emergent magnetic field **b**(**k**) near a Weyl point. The flux penetrating the area *S* is given by the solid angle subtended by *S*, and the total flux is 2*πη*. Therefore, Weyl fermion acts as a monopole or anti-monopole of the emergent magnetic field depending on the chirality *η*=±1.
